# What is the optimum incision for superficial temporal artery biopsy? An anatomical study using body donors

**DOI:** 10.1007/s00276-026-03895-x

**Published:** 2026-05-07

**Authors:** Oliver Vij, Chee Yen Soon, Christopher Smith, Malcolm Neil, Sarah Fawcett, Cecilia Brassett

**Affiliations:** 1 Faculty of Dentistry, Oral and Craniofacial Sciences, King’s College London, Guy’s Hospital, Great Maze Pond, London, SE1 9RT UK; 2 Human Anatomy Centre, Department of Physiology, Development and Neuroscience, University of Cambridge, Cambridge, UK

**Keywords:** Superficial temporal artery, Temporal artery biopsy, Giant cell arteritis, Facial nerve injury

## Abstract

**Purpose:**

Superficial temporal artery (STA) biopsy remains an important diagnostic investigation for giant cell (temporal) arteritis. Incisions should optimise STA exposure while avoiding the temporal branch of the facial nerve. However, the widely used Gillies incision, a 2 cm temporal incision 2.5 cm anterior and superior to the auricular helix within the hairline, has been shown to be inconsistent for STA access.

**Methods:**

In this study of 20 hemifaces from 10 body donors (mean age 87.1; range 75–93; n = 3 females), STA branches were mapped using a Cartesian grid referenced to the anterior crus-lateral canthus axis. Four 2 cm incisions were modelled: Gillies, pre-auricular, and two novel algorithmically optimised incisions targeting frontal and parietal branches. Access was defined as incision-to-vessel distance of ≤ 0.5 cm or ≤ 1.0 cm. All donors had provided written consent for anatomical research under the Human Tissue Act 2004.

**Results:**

The Gillies incision accessed the frontal branch in ≤ 1.0 cm in 11/20 (55%) and ≤ 0.5 cm in 3/20 (15%); parietal access was ≤ 1.0 cm in 8/19 (42%) and ≤ 0.5 cm in 4/19 (21%). Pre-auricular incision improved access: ≤ 1.0 cm for frontal 13/20 (65%) and parietal 18/19 (94.7%) branches. Optimised frontal and parietal incisions achieved ≤ 1.0 cm access in 19/20 (95%) and 18/19 (94.7%) respectively.

**Conclusion:**

Our findings suggest that the Gillies incision may not be a reliable approach for accessing the frontal or parietal branches of the STA. Pre-auricular and algorithmically optimised frontal and parietal incisions achieved high, branch-specific access but require clinical validation.

**Supplementary Information:**

The online version contains supplementary material available at 10.1007/s00276-026-03895-x.

## Introduction

Giant cell (temporal) arteritis is a medical emergency since delayed treatment can lead to occlusion of cranial blood vessels resulting in blindness or stroke [15, 25]. Although diagnostic pathways have evolved and vascular ultrasound is the recommended first-line imaging modality in patients with suspected cranial giant cell arteritis, temporal artery biopsy retains an important role when imaging is unavailable, inconclusive, or when histological confirmation is required [4, 18]. Reliable biopsy technique therefore remains clinically relevant.

Temporal artery biopsy must achieve an adequate arterial specimen while minimising operative morbidity. This is important because false-negative biopsy results may arise from inadequate specimen length, tissue contraction and skip lesions [10]. Currently there is no consensus on minimum specimen length, with the British Society of Rheumatology and American College of Rheumatology both recommending biopsy specimens of at least 10 mm [15, 18]. The ability to identify the superficial temporal artery (STA) consistently is therefore central to both diagnostic yield and procedural efficiency.

The frontal branch of the STA is the traditional target for biopsy [19]. When accessing the frontal branch of the STA, the temporal branch of the facial nerve (TFN), which innervates orbicularis oculi and frontalis muscles, is at risk of damage [24]. It is therefore essential that an approach to the STA avoids iatrogenic damage to the TFN [19]. The area where the TFN is most at risk is described as the anatomical ‘danger zone’, in which the TFN and frontal branch of STA are only separated by the superficial temporal fascia [30]. This ‘danger zone’ is marked as the area inferior to the line between the tragus of the ear and 2 cm above the superior orbital rim, also known as Pitanguy’s line [30]. Despite this knowledge, variability in the TFN can make it vulnerable to iatrogenic injury when using the frontal branch of the STA to obtain a biopsy [24].

Alternative biopsy approaches have therefore been proposed. Posterior or hairline incisions may reduce the risk of TFN injury while preserving specimen quality, and recent clinical and educational reports have renewed interest in biopsy of the parietal branch of the STA which lies outside of the anatomical danger zone [3, 6, 19]. The Gillies incision has also been proposed for access to either the parietal or frontal branches of the STA because it lies within the hairline and away from the anatomical danger zone. This is a 2 cm temporal incision 2.5 cm superior and anterior to the helix of the ear [17]. Although primarily used for indirect access to the zygomatic arch, it may be attractive for STA biopsy because it lies within the hairline and avoids both facial scarring and the anatomical danger zone. Reliable landmark-based incision placement remains important since giant cell arteritis may reduce arterial flow, limiting Doppler localisation, and ultrasound-guided biopsy does not improve sensitivity [8, 17].

The reliability of the Gillies incision has not yet been investigated anatomically to identify whether both parietal and frontal branches of the STA are frequently accessible with this technique. A previous radiological study has suggested considerable variability in the position of the STA bifurcations, making this an unreliable approach [9]. We therefore evaluated the relationship between the STA and four biopsy incisions in a cohort of body donors: a Gillies incision, a pre-auricular incision, and two novel algorithmically optimised branch-specific incisions targeting the frontal and parietal branches respectively. We hypothesised that the Gillies incision would show inconsistent anatomical access to the STA branches, whereas more inferior or posteriorly based incisions would provide more reliable access to the STA.

## Materials and methods

### Study design and ethical approval

A paired-side body donor anatomical study was conducted on 10 formalin-fixed adult body donors (20 hemifaces). All specimens were free of visible scalp or temporal artery pathology and shaved to clearly delineate external landmarks. Methodological planning followed the AQUA and CACTUS guidelines for anatomical and body donor research [16, 27]. All donors had been prepared using an arterial embalming technique by cannulation of the common carotid or femoral artery, and injection under pressure of a solution containing 38% ethanol, 1.5% methanol, 4.2% formaldehyde and 56.3% distilled water. All donors had provided written consent for the use of their bodies for anatomical research, in compliance with the Human Tissue Act 2004. Ethical approval for the study use of donor material was granted by the Human Anatomy Centre, Department of Physiology, Development and Neuroscience, University of Cambridge (Human Tissue Authority Anatomy Licence Number: 12146) on 18/11/2024. The study adhered to the UK Human Tissue Authority guidelines and the Declaration of Helsinki [11, 29].

**Fig. 1:**
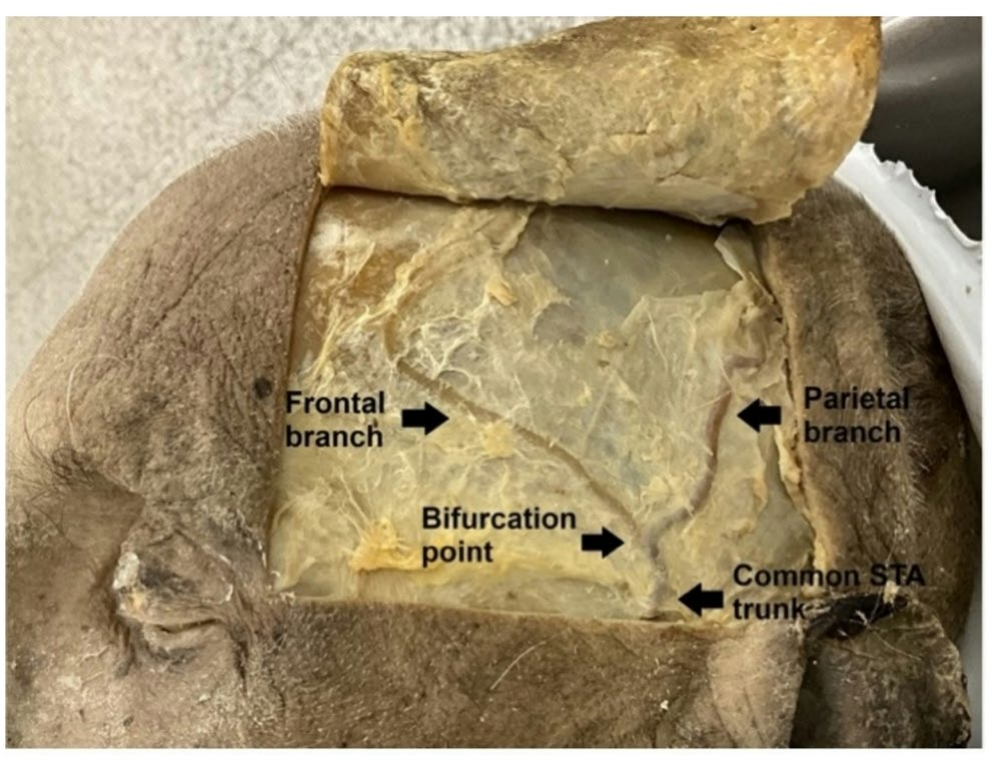
Dissection of STA with flap reflected cranially

Heads were positioned in the Frankfurt horizontal plane [21]. On each side, a horizontal incision was made from the anterior crus of the helix to the lateral canthus to form the x-axis. Vertical releasing incisions were made at each end of the incision and reflected cranially (Fig. 1).

The superficial temporal artery (STA) was dissected and left in situ. The trunk, bifurcation, and frontal and parietal branches were mapped relative to the reference axes. The site of a potential Gillies incision was explored deep to the superficial temporal fascia to identify whether the TFN was present in this area.

### Data collection

A right-handed 2D Cartesian co-ordinate system was established with the x-axis as the straight line joining the anterior crus of the helix (0,0) to the lateral canthus. The y-axis was perpendicular to the x-axis, with positive values cranial to the axis. Coordinates were recorded using callipers and a steel rule. In specimens where the bifurcation lay caudal to the x-axis, the points at which the frontal and parietal branches crossed the x-axis were recorded instead. For pooled analysis, left-sided data were mirrored into a common right-sided coordinate frame so that all coordinates could be analysed relative to a standardised reference system.

Co-ordinates were entered into Microsoft Excel Version 16.105.2 (Microsoft Corporation) and converted to centimetres for analysis.

### Statistical analysis

Four 2 cm incisions were evaluated: a standard Gillies incision, a vertical pre-auricular incision, and two novel algorithmically optimised incisions targeting the frontal and parietal branches respectively. For pooled analyses, left-sided data were mirrored into a common right-sided Euclidean reference frame before incision modelling. Each candidate incision was represented as a closed finite 2 cm line segment within this coordinate system, and vessel course was approximated by a finite straight-line segment joining the recorded trunk point to the recorded frontal or parietal branch point within the dissection window. Hemifaces without a recordable frontal or parietal branch segment were excluded from branch-specific analyses, and denominators are reported throughout.

For an incision segment and a vessel segment, the minimum incision-to-vessel distance was defined as the shortest Euclidean segment-to-segment distance. This was zero when the two segments intersected; otherwise, it was taken as the minimum of the relevant endpoint-to-segment distances. A vessel was considered “accessed” if this minimum distance was at most 1.0 cm or 0.5 cm. These thresholds were selected as pragmatic anatomical definitions of potential vessel encounter and were applied uniformly across all comparator incisions. Primary outcomes were frontal and parietal branch access rates for each incision at both thresholds.

Access proportions were reported with Wilson 95% confidence intervals. Since multiple incisions were evaluated on the same hemiface, paired comparisons of binary access outcomes between incisions were performed using exact McNemar’s tests at r = 1.0 cm and r = 0.5 cm. All tests were two-sided.

A constrained deterministic optimisation was then performed to identify frontal- and parietal-targeted 2 cm incisions. For the frontal-optimised incision, candidate incisions were restricted to a 45° trajectory; for the parietal-optimised incision, candidate incisions were restricted to a 135° trajectory. Candidate midpoints were evaluated over a discretised lattice spanning the bounding rectangle of all pooled STA co-ordinates, expanded by 1 cm in all directions. At each lattice point, access rates were computed across all eligible hemifaces. Candidate solutions were ranked using a predefined lexicographic objective hierarchy. For the frontal-optimised incision, the primary objective was maximal frontal branch access; ties were resolved by higher either-branch access, lower mean distance to frontal branch segments, and lower vertical dispersion among frontal hits. Equivalent rules were applied for the parietal-optimised incision using parietal branch access as the primary objective. The reported solution therefore represents the global optimum within the constrained search space under the specified objective ordering. Full computational methods are provided in Supplementary Appendix A.

All statistical analyses and graphics were generated using RStudio Version 2025.05.1 + 513. As the optimisation procedure was deterministic rather than stochastic, the identified optimal incisions are fully reproducible given the same input data and parameter constraints.

### Sample size

Power analysis using G*Power (version 3.1.9.6) indicated that 8 body donors would provide 80% power to detect a 1 cm STA trunk position difference (SD 0.8 cm, two-tailed α = 0.05). We therefore included 20 hemifaces from 10 body donors, giving 94% power, comparable to previous body donor studies [22].

## Results

We analysed 20 hemifaces in 10 body donors (3 female, 7 male; mean age 87.1 years, range 75–93).

### Right side

**Fig. 2:**
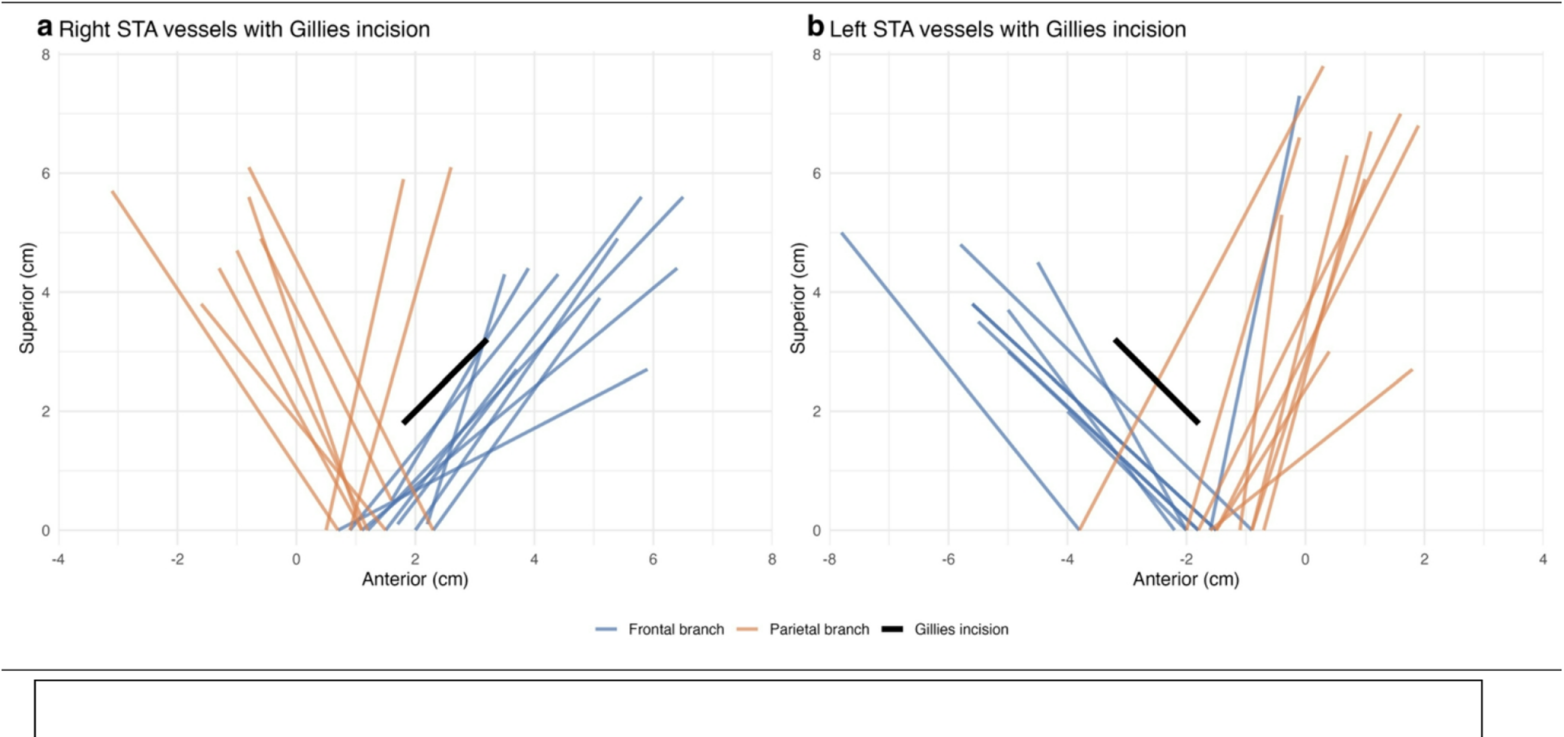
Distribution of frontal and parietal branches of STA with Gillies incision for right (**a**) and left (**b**)

On the right, 30% (3/10; 95% CI 10.8–60.3) of frontal branches lay within 0.5 cm of the Gillies incision and 80% (8/10; 95% CI 49.0–94.3) within 1 cm. For the parietal branch, 22.2% (2/9; 95% CI 6.3–54.7) lay within 0.5 cm and 44.4% (4/9; 95% CI 18.9–73.3) within 1 cm (Fig. 2a). The TFN was not encountered.

### Left side

On the left, 0% (0/10; 95% CI 0.0–27.8) of frontal branches lay within 0.5 cm of the Gillies incision and 30% (3/10; 95% CI 10.8–60.3) within 1 cm. For the parietal branch, 20% (2/10; 95% CI 5.7–51.0) lay within 0.5 cm and 40% (4/10; 95% CI 16.8–68.7) within 1 cm (Fig. 2b). The TFN was not encountered.

### Combined analysis

In pooled analysis, frontal branches clustered within a relatively narrow anterosuperior field from the anterior crus of the helix, whereas parietal branch position showed broader anterior–posterior variability. At r = 1.0 cm, the Gillies incision accessed the frontal branch in 55.0% (11/20; 95% CI 34.2–74.2) and the parietal branch in 42.1% (8/19; 95% CI 23.1–63.7). At r = 0.5 cm, Gillies accessed the frontal branch in 15.0% (3/20; 95% CI 5.2–36.0) and the parietal branch in 21.1% (4/19; 95% CI 8.5–43.3). These results are summarised in Table 1.

**Table 1:** Relationship of frontal and parietal branches of STA to Gillies incision for each side and combined

		Within 0.5 cm of Gillies Incision	Within 1 cm of Gillies Incision
Left side	Frontal	0% (95% CI 0.0–27.8)	30% (95% CI 10.8–60.3)
	Parietal	20% (95% CI 5.7–51.0)	40% (95% CI 16.8–68.7)
Right side	Frontal	30% (95% CI 10.8–60.3)	80% (95% CI 49.0–94.3)
	Parietal	22.2% (95% CI 6.3–54.7)	44.4% (95% CI 18.9–73.3)
Combined	Frontal	15% (95% CI 5.2–36.0)	55% (95% CI 34.2–74.2)
	Parietal	21% (95% CI 8.5–43.3)	42% (95% CI 23.1–63.7)

### Proposed optimised incisions

Algorithmically optimised incisions were identified for both the frontal and parietal branches. These novel incisions will be referred to as optimised frontal incision and optimised parietal incision respectively.

The optimised frontal incision was a 45° incision centred at 2.25 cm anterior and 0.75 cm superior to the anterior crus of the helix. This incision reached the frontal branch in 95% (19/20) of specimens for both r = 0.5 cm (95% CI 76.4–99.1) and r = 1.0 cm (95% CI 76.4–99.1) with a median separation of 0.055 cm. The optimised frontal incision also accessed the parietal branch in 94.7% (18/19; 95% CI 75.4–99.1) of specimens for r = 1.0 cm and 68.4% (13/19; 95% CI 46.0–84.6) of specimens at r = 0.5 cm.

The optimised parietal incision was a 135° incision centred at 0.40 cm anterior and 1.00 cm superior to the anterior crus of the helix. The incision reached the parietal branch in 78.9% (15/19; 95% CI 56.7–91.5) at r = 0.5 cm and 94.7% (18/19; 95% CI 75.4–99.1) at r = 1.0 cm, with a median separation of 0.00 cm. The optimised parietal incision accessed the frontal branch in 80% (16/20; 95% CI 58.4–91.9) of specimens for r = 1.0 cm and 45% (9/20; 95% CI 25.8–65.8) of specimens with r = 0.5 cm.

A vertical pre-auricular incision of 2 cm was evaluated, starting 0.8 cm anterior to the anterior crus of the helix (0,0) [1]. Using this incision, the frontal branch lay within 0.5 cm in 30% (6/20; 95% CI 14.5–51.9) and within 1.0 cm in 65% (13/20; 95% CI 43.3–81.9) of specimens (median separation ~ 0.75 cm); the parietal branch lay within 0.5 cm in 89.5% (17/19; 95% CI 68.6–97.1) and within 1.0 cm in 94.7% (18/19; 95% CI 75.4–99.1), with a median separation of 0.00 cm. This is summarised in Table 2.

**Table 2:** Combined results showing the relationship of the frontal and parietal branches of the STA to a pre-auricular incision, optimised frontal and parietal incisions

		Pre-auricular incision	Optimised frontal incision	Optimised parietal incision
Frontal	r = 0.5 cm	30% (95% CI 14.5–51.9)	95% (95% CI 76.4–99.1)	45% (95% CI 25.8–65.8)
	r = 1.0 cm	65% (95% CI 43.3–81.9)	95% (95% CI 76.4–99.1)	80% (95% CI 58.4–91.9)
Parietal	r = 0.5 cm	89.5% (95% CI 68.6–97.1)	68.4% (95% CI 46.0–84.6)	78.9% (95% CI 56.7–91.5)
	r = 1.0 cm	94.7% (95% CI 75.4–99.1)	94.7% (95% CI 75.4–99.1)	94.7% (95% CI 75.4–99.1)

**Fig. 3:**
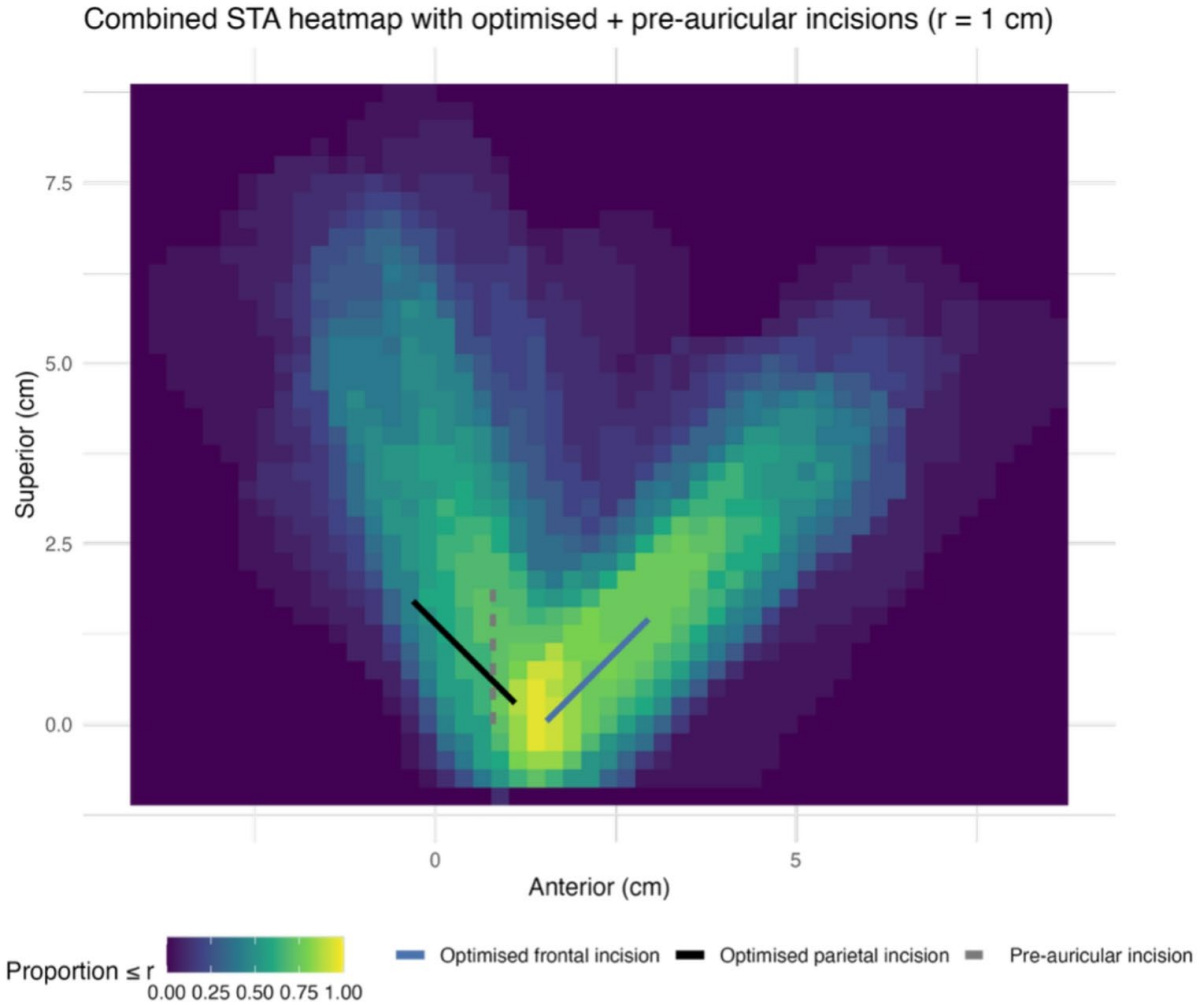
Heatmap (with r = 1 cm) of distribution of frontal and parietal branches of STA with pre-auricular and optimised incisions overlaid

Compared with Gillies, the pre-auricular incision significantly improved parietal access (94.7% vs 42.1% within 1.0 cm; exact McNemar *p* = 0.006), but the improvement in.

frontal access (65.0% vs 55.0% within 1.0 cm) was not statistically significant (*p* = 0.73). Optimised incisions improved target-branch access compared with Gillies (optimised frontal incision: 95.0% vs 55.0% within 1.0 cm; *p* = 0.008; optimised parietal incision: 94.7% vs 42.1% within 1.0 cm; *p* = 0.006). Figure 3 shows a heatmap of the distributions of the frontal and parietal branches of the STA with the optimised frontal incision, optimised parietal incision and pre-auricular incisions overlaid.

## Discussion

Clinically, accurate identification of the STA can be challenging: palpation can be unreliable, particularly in patients with significant subcutaneous tissue or underlying vascular pathology, and Doppler may not always provide definitive vessel localisation [13]. Therefore, a standardised, anatomically reliable incision site could be useful to improve diagnostic yield and minimise complications.

In this body donor anatomical study, the Gillies incision showed inconsistent access to the STA and performed poorly for both frontal and parietal branch localisation, whereas the pre-auricular and algorithmically optimised incisions showed substantially higher access rates. The optimised parietal incision produced the highest parietal branch access, while the pre-auricular incision provided the most reliable dual-branch access overall. These findings support the hypothesis that the Gillies incision is less reliable than more inferior or posteriorly based approaches for locating the STA.

The Gillies incision lay within 1 cm of the frontal branch in only 55% of specimens and of the parietal branch in 42.1%, indicating inconsistent access to either vessel. This supports previous radiological work showing substantial variability in STA bifurcation and helps explain why the Gillies incision may perform poorly across patients [9]. Recent work by Dissaux et al. showed that CT angiography can reproducibly characterise several clinically relevant STA parameters, including termination pattern, branch orientation and relationships to superficial landmarks [5]. Although our study was based on body donors and focused on landmark-based incision planning, the radiological reproducibility demonstrated by Dissaux et al. complements our findings and supports future correlation of imaging with surface landmarks for patient-specific biopsy planning.

By contrast, both the pre-auricular and algorithmically optimised incisions provided significantly better vessel access than the Gillies incision. Both the pre-auricular and optimised parietal incisions achieved 94.7% parietal branch access within 1 cm. The optimised frontal incision was able to access both branches, with 95% accessing the frontal branch and 94.7% accessing the parietal branch within 1 cm. Our algorithmically optimised frontal incision must be interpreted cautiously because its location is closer to the anatomical danger zone defined by Pitanguy’s line and therefore may carry greater risk to the TFN despite its high dual-branch access rate.

These findings also highlight that incision planning for temporal artery biopsy should not be judged on vessel encounter alone [20]. A more anterior or frontal approach may increase access to the frontal branch but may simultaneously bring the surgeon closer to the temporal branch of the facial nerve, which is the principal morbidity concern in this procedure. Previous anatomical and clinical literature has shown that facial nerve injury, including transient or persistent brow dysfunction, is a recognised complication of frontal-branch biopsy approaches [24, 30]. Accordingly, the apparent performance advantage of the frontal-optimised incision must be interpreted in the context of this anatomical trade-off, and this is why the parietal-optimised incision may be more attractive clinically despite slightly lower frontal branch access.

Among the tested approaches, the optimised parietal incision may represent the most clinically pragmatic when broader vessel access is desired. Although its frontal branch access was inferior to that of the optimised frontal incision, it provided excellent parietal branch access and broader dual-branch exposure than the Gillies and pre-auricular incisions. This may be advantageous in temporal artery biopsy when wider exploration is required, although this would need confirmation in clinical studies.

Our findings are consistent with existing literature questioning the reliability of the Gillies approach and support a more inferior or posteriorly based strategy for vessel localisation. The optimised parietal incision is concordant with recent body donor evidence identifying a reliable STA encounter point near the anterior crus of the helix and favouring a posterior-cranial oblique incision trajectory [7]. Although the methodology of Engelmann et al. differed from ours, the directional agreement between the two studies strengthens the argument that a more posteriorly based incision may be anatomically more reliable than a fixed superior hairline approach [7].

This interpretation is further supported by recent clinical and educational reports advocating biopsy of the parietal branch outside the frontal danger zone. Czyz et al. found that more posterior incision placement reduced the risk of motor nerve injury without compromising specimen quality, while Osei et al. argued that parietal-branch biopsy is more teachable and safer for trainees because it avoids the area where the temporal branch of the facial nerve is most vulnerable [3, 19]. Elahi et al. likewise described the parietal branch as a viable and well-tolerated alternative target in the diagnosis of giant cell arteritis [6]. Taken together with the present findings, these studies suggest that posterior or parietal strategies may offer a more favourable balance between vessel accessibility and procedural safety than traditional frontal or hairline approaches.

Aesthetic considerations also affect incision choice. While the Gillies incision may conceal the scar within the hairline, this advantage may be less marked in the older biopsy population because hair thinning and recession can expose the scar [23]. Older patients often have well-developed relaxed skin tension lines, which may improve scar healing and visibility, making the pre-auricular or parietal-specific incisions a viable aesthetic alternative [2].

### Limitations

As with many body donor anatomical studies, donor availability constrained the sample size to 20 hemifaces in 10 body donors, which may not capture the full anatomical variability of the living population, particularly across different ages and ethnicities. Accordingly, this study was designed as an anatomical proof-of-concept and the findings should be interpreted with appropriate caution [12].

Vessel course was simplified as straight-line segments for modelling, and formalin fixation may have altered tissue dimensions and arterial calibre. Multiple studies show that formalin fixation (and subsequent processing) produces measurable shrinkage, typically on the order of approximately 5–20% depending on tissue, fixation time, and processing steps [26, 28]. This can bias absolute distances and lead to underestimates of true in-vivo sizes. In addition, fixation stiffens soft tissues and reduces arterial lumen calibre, potentially modifying the apparent course or accessibility of branches during dissection [14]. We did not apply a universal correction factor because shrinkage is tissue- and protocol-dependent; instead, we anchored all measurements to a constant landmark (anterior crus of the helix) and reported tolerance-based access metrics (≤ 0.5–1.0 cm). Nevertheless, some degree of systematic underestimation is likely, and in-vivo validation would help calibrate these body donor measurements.

## Conclusion

Our findings suggest that the Gillies incision may not be a reliable approach for accessing the frontal or parietal branches of the STA. While the pre-auricular incision provides broader vessel access and potentially higher diagnostic yield, its proximity to the facial nerve necessitates accurate knowledge of anatomical landmarks. Our algorithmically optimised frontal and parietal incisions achieved high branch-specific access. However, the frontal-optimised incision may lie closer to the TFN danger zone and therefore requires clinical validation before routine adoption.

## Supplementary Information

Below is the link to the electronic supplementary material.

Supplementary Material 1

## Data Availability

Data available on request. If any dataset elements cannot be shared publicly due to governance requirements relating to donated human material, those elements will be made available subject to approval by the relevant institutional authority.
