# Supplementary material for: What is the optimum incision for superficial temporal artery biopsy? An anatomical study using body donors
Source: Surg Radiol Anat. 2026 May 7;48(1):123. doi: 10.1007/s00276-026-03895-x (PMC13152922; doi:10.1007/s00276-026-03895-x)
Supplement: Supplementary file 1 — Supplementary Material 1 [file 276_2026_3895_MOESM1_ESM.docx]

Appendix 1: Detailed methods of the statistical and algorithmic analysis used to calculated the optimised incisions.

### **Statistical and Algorithmic Analysis**

For combined analyses, left-sided data were mirrored across the Y-axis (x = -x, y = y) to express all co-ordinates in a common right-ear frame.

Incision parameterisation

Any candidate skin incision was represented as a 2.0 cm line with midpoint C = (c_x_,c_y_) and angle θ (° from the +x axis). With half-length L = 1.0 cm and unit direction u = (cosθ, sinθ), endpoints are as follows:

- A = C – L_u_
- B = C + L_u_

Comparator incisions were defined beforehand:

- Gillies incision:
  - Right: midpoint (2.5,2.5), θ = 45°
  - Left: midpoint (-2.5,2.5), θ = 135°
- Pre-auricular vertical:
  - x = 0.8
  - From y = 0.0 to y = 2.0

Segment-segment distance and “access” definition

For an incision segment AB, and a vessel segment TQ, the segment-segment distance d(AB, TQ) was:

- d = 0 if segments intersect; otherwise
- The minimum of four point-to-segment distances:
  - d(A, TQ), d(B, TQ), d(T, AB), d(Q, AB).

A vessel was counted as “accessed” if the minimum segment-segment distance d(AB, TQ) was ≤ r, with r = 1.0 cm and r = 0.5 cm. A vessel segment was defined as a finite straight line that approximates the course of a branch within the dissection window. For each hemi-face we joined the recorded trunk point T to the recorded branch point Q. For frontal vessel segments this is TF and for parietal vessel segments this is TP (each a straight segment TQ). Hemi-faces without a recorded frontal or parietal point were excluded from analyses for that branch (denominators reported).

Outcomes:

For each incision we calculated:

1. Frontal branch access rate: proportion of specimens with d(AB, T→F) ≤ r
2. Parietal branch access rate: proportion of specimens with d(AB, T→P) ≤ r

For each incision and branch, access proportions were reported with Wilson 95% confidence intervals. Because multiple incisions were evaluated on the same hemi-face, paired comparisons of access (accessed vs not accessed) between incisions were performed using exact McNemar’s tests at r = 1.0 cm and r = 0.5 cm. Minimum incision-to-vessel distances were additionally summarised as median (IQR) for each incision.

Algorithmic optimisation of incision location:

We performed a deterministic grid search for two clinically constrained incision families.

- A frontal-optimised set: θ = 45°
- A parietal-optimised set: θ = 135°

Search region:

We computed the bounding rectangle of all STA co-ordinates and expanded it by 1 cm in all directions. Candidate midpoints were evaluated on a lattice with 0.25 cm spacing (checked at 0.10 cm in sensitivity).

Scoring:

At each grid point C, endpoints A,B were generated and d(AB, T→F) and/or d(AB, T→P) were computed for every specimen. We then formed access rates at radius r (above).

Selection:

- For frontal-optimised incisions at 45°, the primary objective was maximising frontal access rate. Ties were broken, in order, by: (i) higher either-branch access, (ii) lower mean distance to frontal segments, (iii) lower vertical dispersion (variance of y among frontal hits).
- For parietal-optimised incisions at 135°, the same rules were applied with parietal as the target.

The chosen midpoints were then reported with their 2 cm endpoints (via the parameterisation above). These are the “algorithmically optimised incisions” used in figures and tables.

All statistical analysis and graphics were generated using RStudio Version 2025.05.1+513. No stochastic methods were used meaning the optimisation is fully reproducible given the data and parameters above.
